# Intermediate-term outcome of cryoballoon ablation of persistent atrial fibrillation and improvements in quality of life of patients

**DOI:** 10.1371/journal.pone.0261841

**Published:** 2022-01-21

**Authors:** Daniel Mol, Anchee M. Boersma, Wouter R. Berger, Muchtiar Khan, Gijsbert S. de Ruiter, Geert-Jan P. Kimman, Joris R. de Groot, Jonas S. S. G. de Jong

**Affiliations:** 1 Department of Cardiology, OLVG, Amsterdam, the Netherlands; 2 Department of Cardiology, Amsterdam University Medical Centres, Amsterdam, the Netherlands; 3 Department of Cardiology, Noord-West Ziekenhuisgroep, Alkmaar, the Netherlands; University of Bologna, ITALY

## Abstract

**Background:**

Clinical outcome of pulmonary vein isolation (PVI) has been predominantly focused on the reoccurrence of atrial fibrillation (AF) and the maintenance of sinus rhythm. However, there has been a limited intermediate follow-up on health-related quality-of-life (HRQoL) of patients. Given the relatively high recurrence rate of persistent AF after PVI treatment, it is important to follow up with clinical outcomes on symptom improvement as well as health-related quality-of-life. This study was designed to investigate the recurrence rate of atrial tachyarrhythmia (ATa), AF-related symptoms and HRQoL after PVI in patients with persistent AF who were treated with the second generation cryoballoon.

**Methods:**

Total 148 patients participated in the study who were treated by PVI between 2013 and 2017 for persistent AF. All patients visited the out-patient clinic 2–5 years after PVI. During this visit all patients filled-out an AF Effect on Quality-of-life (AFEQT) questionnaire and a seven-day Holter was applied. All rhythm recordings acquired post ablation were collected and analysed, and the modified European Heart Rhythm Association score (mEHRA) scores were calculated before and after the ablation.

**Results:**

The average age of patients was 63±9 year old and 44 (27.9%) were female. Mean CHA_2_DS_2_ VASc score was 1.9±1.4, and moderate and severe left atrial (LA) dilation was present in 53 (36.1%) and 15 (10.2%) patients, respectively. After a follow-up of 3.7±1.0 years, 81 (54.7%) patients had an ATa recurrence and 35 (23.6%) patients underwent a repeat LA ablation. However, the mEHRA score significantly improved in 80.4% of the patients (*p* <0.001), with the median overall AFEQT score of 88.9 [70.4–97.2].

**Conclusions:**

There is a considerable ATa recurrence rate after PVI of persistent AF at intermediate-term follow-up. However, our data indicate that PVI significantly improved AF-related symptoms and resulted in a high HRQoL for 2–5 years in patients with persistent AF.

## Introduction

Catheter ablation of atrial fibrillation (AF) has shown promising outcome in maintaining sinus rhythm and improving health related quality-of-life (HRQoL) [1–3]. Although pulmonary vein isolation (PVI) is the cornerstone of ablation in patients with AF, the rate of freedom of AF after PVI varies among studies, largely depending on the duration of follow-up, the method used for AF monitoring and AF type, as persistent AF is associated with a higher recurrence rate than paroxysmal AF [4, 5].

Patients with persistent AF tend to have a larger left atrial volume, a lower left ventricular ejection fraction and a higher degree of mitral regurgitation that are closely associated with a higher stroke risk [6]. In addition, persistent AF has been associated with an increased incidence of atrial fibrosis [7, 8]. Potential consequences of a more fibrotic left atrium (LA) include the slowed or blocked intra-atrial conduction and the emergence of non-PV ectopic triggers, which may render a PVI-based ablation alone less effective [9]. Indeed, the 2020 European Society of Cardiology (ESC) guideline for diagnosis and management of AF specify that PVI alone may be insufficient for the treatment of persistent AF, and additional ablation strategies may be considered [10]. However, the effectiveness and safety of additional ablation in addition to PVI remain controversial [11].

In addition, 2020 ESC guidelines suggest HRQoL as one of the key outcomes after AF ablation [10]. It recommends to consider patient-oriented outcomes such as HRQoL as an essential factor in the assessment and integrated management of the AF patients [10]. This is likely because arrhythmia-free survival rate alone may not be a sufficient measure of the procedural outcome, as a single event can flip the outcome from success to failure. Therefore, patient-reported outcomes are suggested to add important information for the management of AF patients in our daily practice. Moreover, the high AF recurrence rate even after PVI suggests a very low chance of a permanent cure for AF. Therefore, more attention have been given to the benefits of ablation on HRQoL [10]. However, the evaluation of quality of life after PVI treatment in patients with persistent AF limited to small sized or short-term studies [1, 3, 12].

The second-generation cryoballoon (CB) was introduced with enhanced cooling abilities leading to more sustainable ablation lesions, resulted in improved procedural and mid-term outcomes in comparison with those of the first generation CB [13, 14]. Although various studies have reported outcomes of the second generation CB in patients with persistent AF, data on rhythm and HRQoL outcomes of the second-generation CB therapy are limited to short-term follow-ups [15–20]. In addition, it remains unclear if PVI, the major ablation strategy for patients with persistent AF, is sufficient to achieve a clinically satisfactory outcome in terms of freedom of AF, reduction of AF related symptoms and HRQoL.

Accordingly, we here investigated the outcomes of second-generation CB in patients with persistent AF for 2–5 years, with emphasis on arrhythmia recurrence, HRQoL and improvement of symptom scores after the ablation procedure.

## Methods

### Study participants

A cross-sectional single centre study was conducted. Briefly, consecutive patients with drug-refractory persistent AF who underwent PVI between 2013 and 2017 in OLVG Hospital Amsterdam, the Netherlands, were approached for participation. Persistent AF was defined as at least one AF episode lasting over 7 days but less than 365 days. Patients eligible for the study 1) had persistent AF, 2) had no previous AF ablation, 3) were treated with the second-generation CB with PVI without additional left atrial ablation, and 4) signed on informed consent. The study was conducted in full accordance with the principles of the "Declaration of Helsinki" (64^th^ WMA General Assembly, Fortaleza, Brazil, October 2013). The study was approved by the Medical Ethics Committee (MEC-U Nieuwegein, the Netherlands). This study was registered in the Dutch trial register *toetsingonline*.*nl* (NL63586.100.17) and data are reported according to the STROBE guidelines.

### Outcome measurements

The primary outcome of the study was the freedom of any atrial tachyarrhythmia (ATa), defined as time to the first documented ATa lasting longer than 30 sec [10]. Secondary outcomes included the freedom of AF, the freedom of atrial tachycardia (AT), the classification of recurrent AF (i.e. paroxysmal, persistent or longstanding persistent), HRQoL during follow-up as per Atrial Fibrillation Effect on Quality-of-life (AFEQT) questionnaire, the documented number of electrical cardioversions (ECV) before PVI and during follow-up, and the number of repeat LA ablations. Safety outcomes were divided into early and late complications. Early complications included vascular complications within 30 days, transient phrenic nerve palsy (PNP) lasting shorter than 24 hours, pericardial effusion occurring within 24 hours and procedural stroke. Late complications included persistent PNP lasting more than 24 hours, pericardial effusion occurrence or persistence between 24 hours and 30 days, and thromboembolic events during 30 days. We further sought to identify relevant clinical variables that are associated with ATa recurrence.

### Echocardiographic definitions

As per guideline recommendations [21, 22], LA dimension and LV function were categorized into normal, mild, moderate or severe enlargement/impairment. If there was no discrete measure available, we used the reported qualitative description of the LA dimension and LV function.

### Ablation procedure

The ablation procedure was performed using the second-generation CB Advance 28-mm (Medtronic, Minneapolis, MN) with uninterrupted oral anticoagulant therapy. For patients who received a vitamin K oral anticoagulant, we used a target prothrombin time at international normalised ratio between 2.0 and 3.0. The ablation procedure was performed under conscious sedation with midazolam, and fentanyl analgesia was administered if necessary. Via femoral vein access, a diagnostic quadripolar catheter was positioned in the coronary sinus, and a transseptal sheath was advanced via a second puncture. After administration of 10.000 IU heparin and obtaining left atrium access, activated clotting time was targeted at 350 sec. A circular octopolar catheter (Medtronic, Minneapolis, MN) was placed in the proximal part of the PV ostium to record PV potentials. The CB was then inflated and advanced into the PV ostium. PV occlusion was confirmed with an injection of contrast dye and ablation was performed for at least 180 seconds. PVI was proven by demonstrating bidirectional conduction block. During the application of cryotherapy to the right PVs, phrenic nerve integrity was continuously monitored through pacing the nerve from the superior vena cava or subclavian vein. After the last application, all PVs were examined for persisting conduction block. If patients were in AF, an ECV was performed before examine the PVs.

### Follow-up

The patients participated in this cross-sectional study were followed up in compliance with standard clinical care with extra visits and rhythm recordings as per physicians’ discretion. If patients remained free of symptoms after two months, we advised the referring physician to discontinue AAD therapy. Patients were encouraged to visit the hospital if they experienced AF-related symptoms. In addition to the routine clinical care follow-up, all patients participating in the study were seen between September 2018 and November 2019, 2–5 years after the index procedure. During this outpatient visit, a twelve-lead ECG was obtained, and a seven-day p-wave centric single lead Holter (BardyDX, Seattle, WA) was performed unless patients had a cardiac implantable device (CIED) allowing detection of ATa. For patients with implanted devices, the device was interrogated prior to the follow-up visit and the seven days preceding interrogation were analysed for the occurrence of atrial arrhythmias related to the current HRQoL. All ATa episodes recorded by CIED were included in arrhythmic follow-up.

In addition, the AFEQT questionnaire was filled out during the visit to evaluated patients’ quality of life related to AF [23]. The AFEQT questionnaire is an AF specific HRQoL score and has been validated previously [23]. It consists of three domains: 1) symptoms, 2) daily activity and 3) treatment concerns, which are consisted with four, eight and six questions, respectively. For each question, an answer can be selected on a 1–7 scale, where one reflects no symptom or limitation and seven represents the most severe symptoms or limitations. The overall score and domain-specific scores were transformed to a 0–100 scale, such that a higher score indicates a higher health status [23]. The transformation formula for this purpose is:

$$score=100-\frac{(the\;sum\;of\;severity\;for\;all\;questions\;answered-number\;of\;question\;answered)*100}{\left(total\;number\;of\;answered\;questions\;*6\right)}$$

Before the procedure and at follow-up visits, we recorded mEHRA score in the medical charts to classify patients’ symptoms as, I: no or mild symptoms, IIa: moderate symptoms but no effect on daily life, IIb: moderate symptoms and daily life affected, III: severe symptoms and IV: disabling symptoms [24].

To document the ATa recurrence, all medical records were retrieved from the referring hospitals and all rhythm recordings obtained after the ablation procedure were analysed. Recurrence of ATa was defined as any documented episode >30sec on any electrocardiographic recording, or spanning the full recording of a standard 12-lead ECG. These also included any ATa recording retrieved from a CIED. We used a 90-day blanking period after each LA ablation. The distribution of types of AF recurrence was described for each follow-up year.

### Statistical analysis

Statistical analysis was performed with R studio (RStudio, Inc., Boston, MA). A sample size calculation was performed estimating at least 95 patients considering a 95% confidence interval to detect an expected ATa recurrence incidence of 55% and a relative precision of 10%. For the collected clinical data, normally distributed continuous variables are presented as mean ± standard deviation. Continuous variables that were not normally distributed were presented as median and interquartile range (IQR). Frequencies are presented as numbers and percentages (%). Missing data, if any, are presented in Table 1. The Wilcoxon signed-rank test, mixed effects models and generalized estimating equations for ordinal multinomial responses were used for repeated measurements. In these models we adjusted for the patient specific follow-up period. Kruskal-Wallis and Dunn’s test were used for multiple comparisons. Multiple test adjustment Bonferroni method was used for p-value adjustment. The Kaplan-Meier curve was used to analyse the time to first event outcomes: ATa, AF and AT recurrence. All relevant clinical variables are assessed in an univariate Cox proportional hazards analysis to identify variables associated with ATa recurrence. Multivariate Cox proportional hazards analysis was performed with associated variables with a p-value < 0.100. Hazard Ratios (HR) are presented with corresponding 95% confidence interval (CI).

**Table 1:** Baseline characteristics of patients.

	Before PVI	Follow-up	
		3.7 ±1.0 years	p-value
			(Before vs at follow-up)
**Age**	62.8 ±8.5	66.5 ±8.3	< 0.001
**Female**	44 (29.7)		
**BMI**	28.3 ±4.8	27.8 ±4.7	0.012
**CHF**	18 (12.2)	22 (14.9)	0.141
**Hypertension**	74 (50.0)	76 (51.4)	0.490
**Diabetes**	7 (4.7)	8 (5.4)	0.032
**Stroke**	16 (10.8)	19 (12.8)	< 0.001
**Vascular disease**	26 (17.6)	30 (20.3)	0.105
**CHA_2_DS_2_ VASc**	1.9 ±1.4	2.1 ±1.4	< 0.001
**Common PV ostium**	22 (14.9)		
***Echocardiography***			
**LA dilation**	(n = 147)		
** None**	25 (17.0)		
** Mild**	54 (36.7)		
** Moderate**	53 (36.1)		
** Severe**	15 (10.2)		
**LV ejection fraction**			
** Normal**	108 (73.0)		
** Mild impaired**	29 (19.6)		
** Moderate impaired**	7 (4.7)		
** Severe impaired**	4 (2.7)		
**Mitral valve regurgitation**			
** None**	69 (46.6)		
** Mild**	52 (35.1)		
** Moderate**	27 (18.2)		
** Severe**	0		
***Rhythm characteristics***			
**mEHRA score**			
** I**	3 (2.0)	76 (51.4)	
** IIa**	17 (11.5)	33 (22.3)	
** IIb**	46 (31.1)	21 (14.2)	
** III**	59 (39.9)	17 (11.5)	
** IV**	23 (15.5)	1 (0.7)	< 0.001
**Implantable cardiac device**	9 (6.1)	14 (9.5)	< 0.001
**Time from AF diagnosis to ablation**	3.7 [1.8–7.3]		
**Number of ECV before ablation**	2.7 ±3.1		
**Previous CTI**	4 (2.7)		
***Medications***			
**Flecainide**	44 (29.7)	6 (4.1)	< 0.001
**Beta-Blockers**	62 (41.9)	44 (29.7)	0.020
**Calcium antagonist**	32 (21.6)	15 (10.1)	< 0.001
**Digoxin**	14 (9.5)	5 (3.4)	0.041
**Sotalol**	31 (21.0)	19 (12.8)	0.011
**Amiodarone**	31 (21.0)	1 (0.7)	< 0.001
**Other AAD**	4 (2.7)	0	NA
**Vitamin-K OAC**	87 (58.8)	39 (26.4)	< 0.001
**Non vitamin-K OAC**	60 (40.5)	69 (46.6)	0.208

*pulmonary vein isolation (PVI), Body mass index (BMI), Congestive heart failure (CHF), CHA_2_DS_2_ VASc (congestive heart failure, hypertension, age (≥75, doubled), diabetes, stroke (doubled), vascular disease, age (≥65years) and sex). Pulmonary vein (PV), left atrium (LA), left ventricle (LV), modified European Heart Rhythm Association score (mEHRA), New York Heart Association class (NYHA), atrial fibrillation (AF), electro cardioversion (ECV), cavotricuspid isthmus ablation (CTI), anti-arrhythmic drugs (AAD), oral anti-coagulants (OAC), new oral anti-coagulant (NOAC). Number (%), mean ± (standard deviation), median [interquartile range].

## Results

### Study population

**Fig 1:**
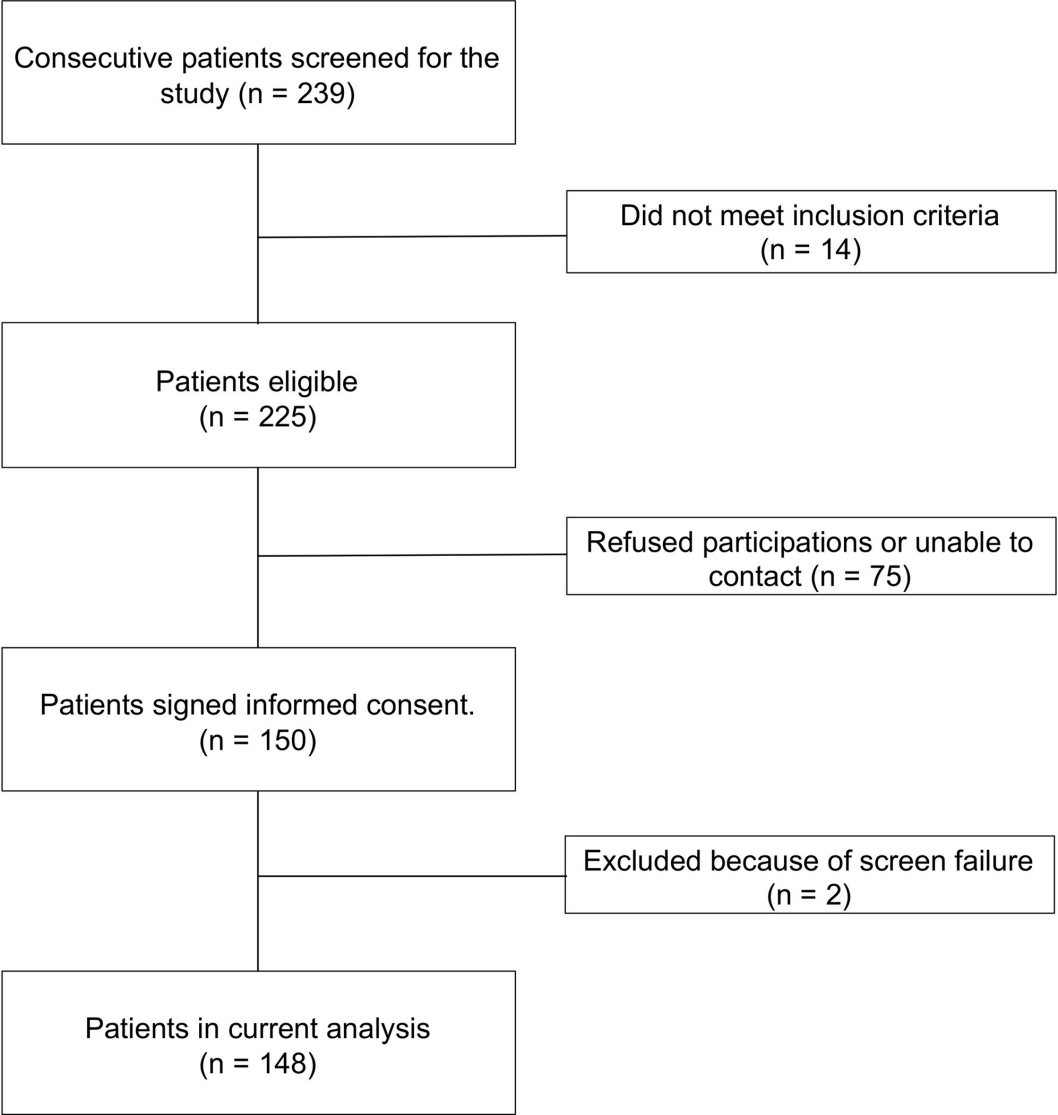
Flowchart of patient selection process.

From total 239 consecutive patients screened, 225 eligible candidates were selected. Seventy-five patients could not be contacted or refused to participate in the study, resulting in total 150 patients signed on informed-consent. Two patients were excluded after signing the informed consent because of screening failure, as one patient was treated with radiofrequency (RF) ablation and the other one received a PVI because of persistent AT. As result, total 148 patients were included in the current analysis (**Fig 1**).

**Table 1** lists the baseline characteristics of participating patients, with the mean age of 63 ±9 year, 104 (82.1%) males and 44 (27.9%) females, and the average body mass index (BMI) of 28.3 ±4.8 kg/m^2^. Mean CHA_2_DS_2_ VASc score was 1.9 ±1.4, and moderate and severe LA dilation was present in 53 (36.1%) and 15 (10.2%) patients, respectively. Thirty-one (21%) and 31 (21%) of patients were under anti-arrhythmic management with amiodarone and sotalol, respectively. Nine (6.1%) patients had a CIED implanted before the PVI. Time from first AF diagnosis to PVI was 3.7 years [IQR 1.8–7.3], 128 (86.5%) patients had an mEHRA score of IIb or higher before the PVI procedure (**Table 1**).

### Procedural and late complications

Eleven (7.4%) patients experienced a procedure-related complication. Transient and persistent PNP occurred in 8 (5.4%) and 1 (0.7%) patients, respectively. There were no thromboembolic events or cases of pericardial effusion within 24h. In 2 patients pericardial effusion occurred after 24h, both cases were treated conservatively. There were no vascular complications requiring surgical intervention, and there were no cases of AV fistula or spurious aneurysms of the femoral vein.

### Clinical and rhythm outcomes at follow-up

Follow-up took place on average 3.7 ±1.0 years after the ablation procedure. At follow-ups, as listed in **Table 1**, mean BMI was lower (27.8 ±4.7 vs 28.3 ±4.8 kg/m^2^, p *=* 0.012) and the CHA_2_DS_2_ VASc score was higher than before the procedure (2.2 ±1.5 vs. 1.9 ±1.4, p < 0.001), mostly driven by the increase of age (before PVI 63 ±9 year vs. 67 ±8 year at follow-up, p < 0.001). Twenty-six (17.6%) patients continued anti-arrhythmic medication at follow-up (flecainide n = 6, sotalol n = 19 and amiodarone n = 1) and a CIED was implanted in 5/139 (3.6%) patients at the follow-up.

**Fig 2:**
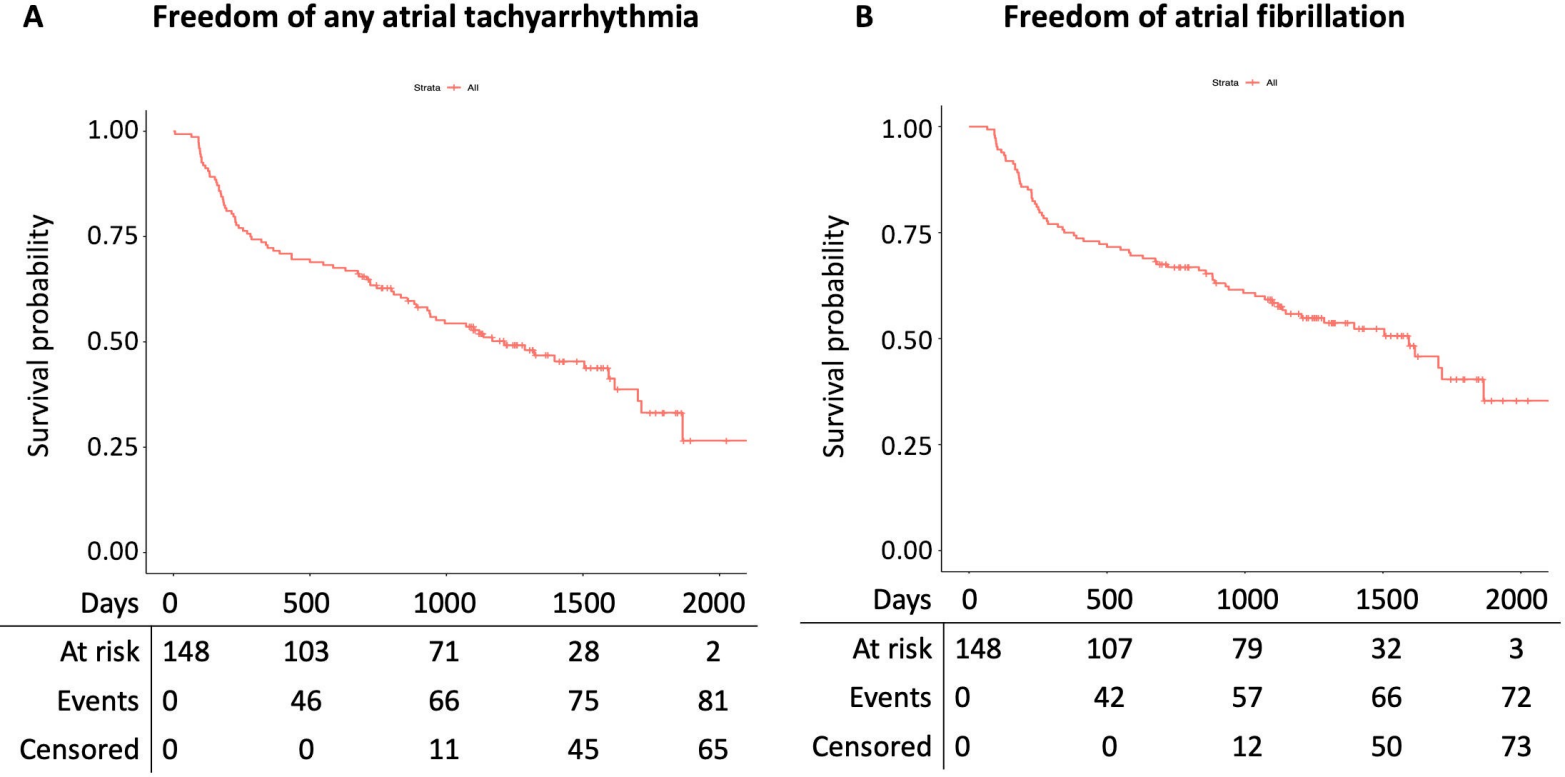
Arrhythmia free survival analyses. A. Survival analysis of freedom of any atrial arrhythmia. B. Survival analysis of freedom of atrial fibrillation.

**Fig 3:**
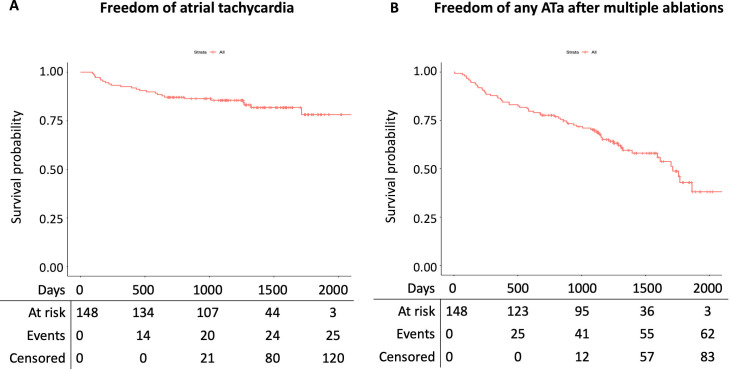
Arrhythmia free survival analyses. A. Survival analysis of freedom of atrial tachycardia. B. Survival analysis of freedom of any atrial arrhythmia after multiple ablations.

In 81 (54.7%) patients, a recurrence of ATa was documented on the follow-up Holter or on the retrieved electrocardiograms. Among the 81 patients with an ATa recurrence, 72 (88.9%) had an AF recurrence, whereas 25 (30.9%) had a recurrence of AT. The corresponding arrhythmia free survival curves of ATa, AF and AT recurrence are presented in **Figs 2** and **3**. In univariate analysis, the presence of hypertension, a mildly or severe impaired LV function, a common PV ostium, a CIED, the use of amiodarone or non-vitamin-K oral anticoagulants were associated with ATa recurrence (p-value < 0.100) (**Table 2**). Multivariate analysis adjusted for these variables showed that patients with mildly impaired LV function had statistical significantly higher ATa recurrence risk (HR 1.76 95% CI 1.02–3.03). In addition, the presence of a common ostium or a CIED demonstrated a non-statistical significant trend towards a higher ATa recurrence risk (adjusted HR 1.76 95% CI 0.99–3.14, and adjusted HR 2.25 95% CI 0.96–5.31, respectively).

**Table 2:** Regression analysis for recurrence of any atrial arrhythmia.

	Hazard Ratio	95% CI	p-value	Adjusted Hazard Ratio	95% CI	p-value
**Female**	1.12	0.70–1.78	0.644			
**Age**	1.01	0.98–1.04	0.616			
**BMI**	1.04	0.99–1.08	0.119			
**Hypertension**	1.63	1.04–2.55	0.032	1.41	0.88–2.27	0.151
**Diabetes**	1.26	0.46–3.46	0.648			
**CHF**	1.04	0.54–2.03	0.899			
**Stroke**	0.64	0.28–1.47	0.290			
**Vascular disease**	0.86	0.48–1.56	0.624			
**CHA_2_DS_2_ VASc**	1.06	0.91–1.24	0.428			
**Common PV ostium**	1.69	0.96–2.97	0.069	1.76	0.99–3.14	0.055
** *Echo—cardiography***						
**LA dilation**						
** None**	REF					
** Mild**	0.87	0.47–1.61	0.648			
** Moderate**	1.12	0.58–2.17	0.735			
** Severe**	1.47	0.74–2.93	0.269			
**LV ejection fraction**						
** Normal**	REF			REF		
** Mild impaired**	2.00	1.18–3.37	0.009	1.76	1.02–3.03	0.043
** Moderate impaired**	0.70	0.22–2.26	0.555	0.52	0.15–1.86	0.314
** Severe impaired**	2.75	0.86–8.86	0.090	2.85	0.86–9.42	0.087
**Mitral valve regurgitation**						
** None**	REF					
** Mild**	0.80	0.48–1.32	0.376			
** Moderate**	1.48	0.84–2.58	0.173			
** Severe**	NA					
** *Rhythm characteristics***						
**mEHRA score**						
** I**	REF					
** IIa**	1.40	0.17–11.19	0.754			
** IIb**	1.72	0.23–12.68	0.594			
** III**	1.51	0.21–11.04	0.687			
** IV**	1.29	0.17–10.00	0.808			
**CIED**	1.15	1.03–5.50	0.042	2.25	0.96–5.31	0.063
**Time from AF diagnosis to ablation**	1.01	0.96–1.06	0.791			
**ECV before ablation**	1.00	0.95–1.06	0.953			
**Previous CTI**	0.75	0.18–3.10	0.690			
** *Medications***						
**Flecainide**	0.76	0.46–1.25	0.283			
**Beta-blockers**	1.22	0.79–1.90	0.371			
**Calcium antagonist**	0.96	0.57–1.61	0.865			
**Digoxin**	1.29	0.67–2.51	0.447			
**Sotalol**	0.80	0.45–1.43	0.454			
**Amiodarone**	1.65	1.01–2.70	0.047	1.39	0.82–2.37	0.220
**Vitamin-K OAC**	1.53	0.96–2.45	0.077	1.50	0.92–2.43	0.101
**Non vitamin-K OAC**	0.68	0.42–1.08	0.103			

Confidence interval (CI), Body mass index (BMI), Congestive heart failure (CHF), CHA_2_DS_2_ VASc (congestive heart failure, hypertension, age (≥75, doubled), diabetes, stroke (doubled), vascular disease, age (≥65years) and sex). Pulmonary vein (PV), left atrium (LA), left ventricle (LV), modified European Heart Rhythm Association score (mEHRA), New York Heart Association score (NYHA), electro cardioversion (ECV), cavotricuspid isthmus ablation (CTI), oral anti-coagulants (OAC).

Repeat LA ablation was performed in 35 (23.6%) patients and in two (1.4%) patients a His ablation was performed during the second ablation procedure. Five (3.3%) patients underwent a third ablation within the study period, including four LA ablations and one His ablation. The median time to first repeat LA ablation was 1.5 years [IQR 0.825–2.756]. Arrhythmia free survival after multiple ablation procedures is shown in **Fig 3B**.

**Fig 4:**
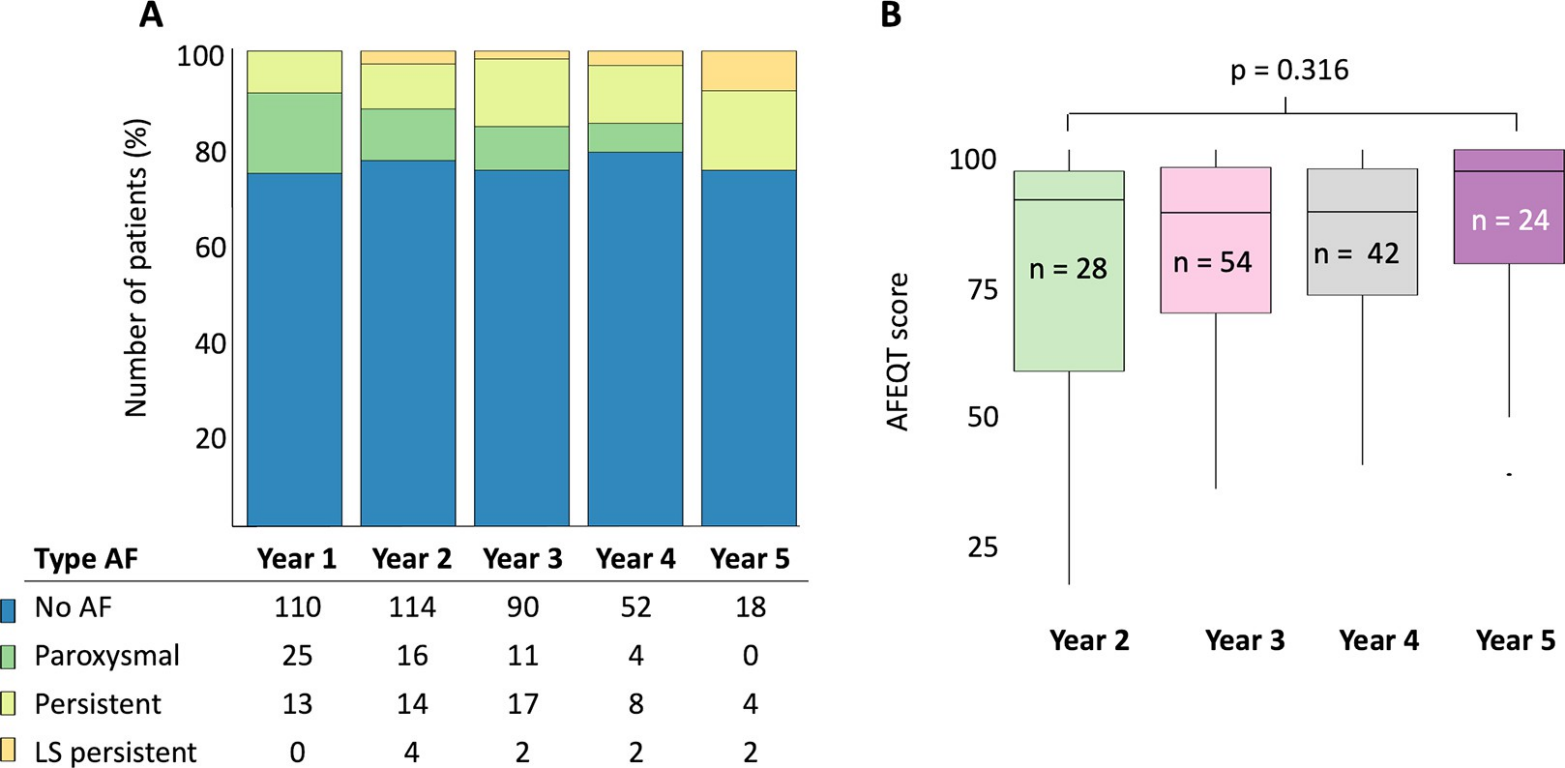
Type of atrial fibrillation per year of follow-up. A. The type of atrial fibrillation (AF) reported for each follow-up year per patient. B. Overall AFEQT score per year. Of note, figure does not display repeated measurements.

**Fig 4A** shows the distribution of the AF type per year follow-up after the ablation procedure. With increasing follow-up duration, the proportion of patients developing persistent or long-standing persistent AF slightly increased. Of the patients with two years of follow-up, 77.0% did not report AF, 10.8% had paroxysmal, 9.5% persistent, and 2.7% long-standing persistent AF. In comparison, at five years of follow-up, 75.0% of patients did not report AF, 0% had paroxysmal, 16.7% persistent, and 8.3% long-standing persistent AF (p = 0.196).

Patients underwent an average number of 0.99 [range, 0.00–15.79] ECVs per year before the PVI procedure, which decreased to 0.27 [range, 0.00–6.17] per year during follow-up (p *<* 0.001). Also, patients with documented recurrence of ATa showed fewer ECVs after the ablation procedure. In those patients, the number of ECVs was 1.07 [range, 0.00–15.79] ECV per year prior, and decreased to 0.50 [range, 0.00–6.17] (p *<* 0.001) after the ablation.

The seven-day Holter registration was completed in all patients without CIED capable of recording atrial arrhythmias (n = 134). Median Holter recording duration was 165.2 [IQR 156.4–169.0] hours, and the median noise burden was 3.9 [IQR 1.4–9.2]%. The Holter recording revealed atrial arrhythmias in 32 (23.9%) patients, including 29 (21.6%) with AF and 5 (3.7%) (also) with AT. In 9 (6.7%) of those patients, the Holter detected recurrence was the first documented recurrence since PVI (AF n = 7, AT n = 1, AF/AT n = 1). In the 14 patients with a CIED, six (42.9%) patients had AF recurrence during the seven days prior to the outpatient clinic visit and 12 (85.7%) patients had an ATa recurrence during the complete follow-up period.

### AF related symptoms and health-related quality-of-life

**Fig 5:**
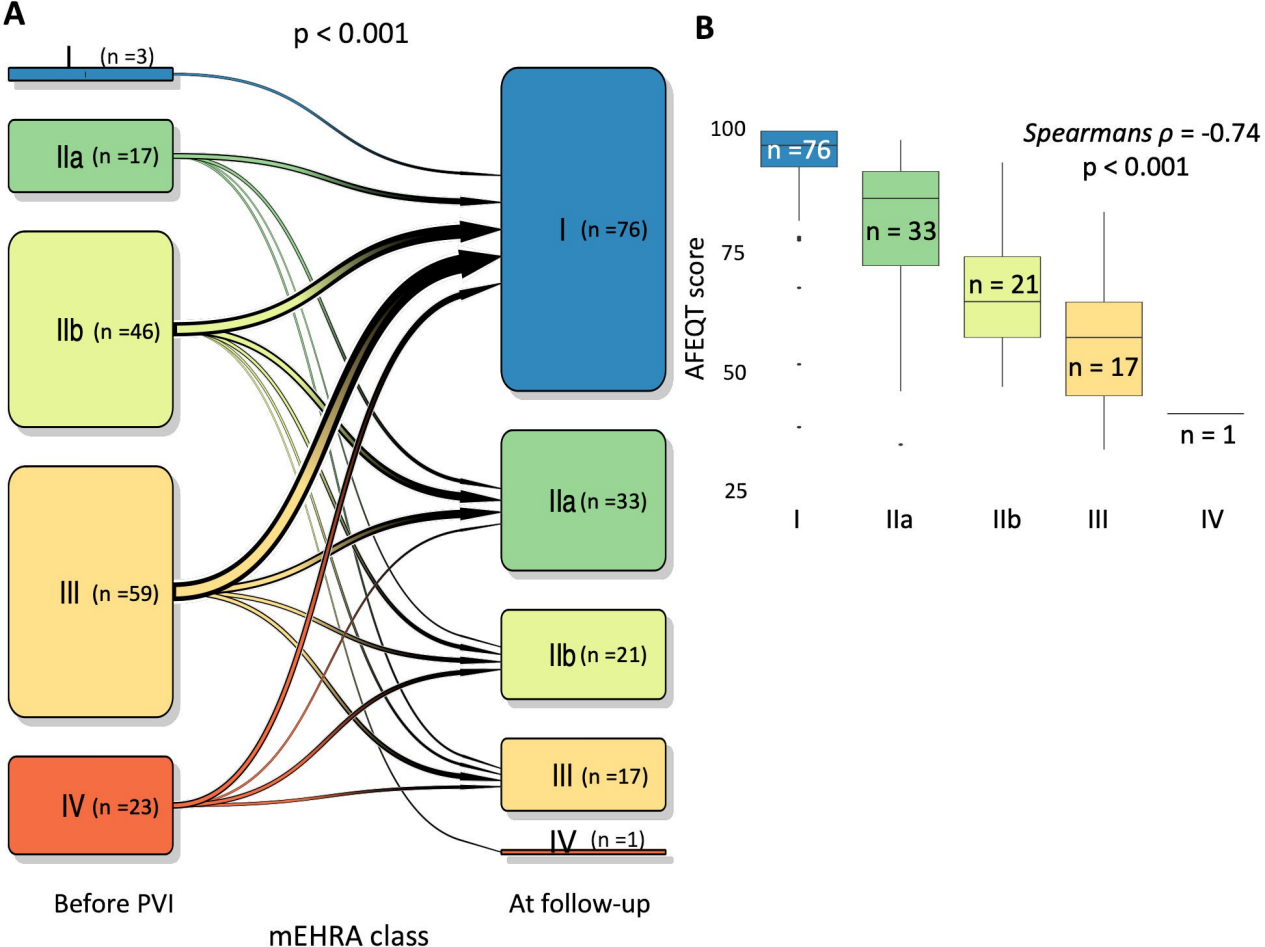
Health-related quality-of-life and AF related symptoms. A. mEHRA score before pulmonary vein isolation (PVI) and at follow-up. The arrow thickness corresponds to the proportion of patients. B. Correlation between mEHRA score and Atrial Fibrillation Effect on Quality-of-life (AFEQT) score at follow-up.

The mEHRA score improved in the majority of patients during the clinical follow-up. Before ablation, 128 (86.5%) patients had an mEHRA score of IIb or higher, while the majority (73.7%) of patients had a score of I or IIa at follow-ups (p < 0.001). One-hundred-nineteen (80.4%) patients had a lower mEHRA class at follow-ups than before the ablation procedure, whereas mEHRA remained unchanged in 22 (14.9%) patients and increased in 7 (4.7%) patients (p *<* 0.001, **Table 1, Fig 5A**). To better describe the extent and nature of AF related symptoms and their influence on the HRQoL, we quantified HRQoL with the AFEQT score at follow-ups. The overall AFEQT quality-of-life score was 88.9 [IQR 70.4–97.2], AFEQT domain symptoms 90.3 [IQR 75.0–100.0], daily activity 89.6 [IQR 59.2–100.0] and treatment concerns 94.4 [IQR 80.6–100.0]. There was a significant correlation between AFEQT score and mEHRA at follow-ups (**Fig 5B**, spearman ρ = -0.74, p < 0.001). The majority of patients with mEHRA class I or IIa at follow-ups had an overall AFEQT score >75 points, whereas the majority of patients with an mEHRA class IIb–IV had an overall AFEQT score ≤75 points.

Patients without ATa recurrence documented on Holter had a higher AFEQT quality-of-life score as compared to those patients with paroxysms or continuous ATa (no AF: 92.7 [IQR 81.7–98.1], AF paroxysms: 75.9 [IQR 56.7–89.1], continuous AF: 70.4 [IQR 51.6–87.7], p *=* 0.013). Lastly, as shown in **Fig 4B**, the overall AFEQT score was equal in patients with 2, 3, 4- or 5-years follow-up although the number of patients with an ATa recurrence increased. Of note, the presented AFEQT scores in this figure are not repeated measurements.

## Discussion

In this cross-sectional real-world study, we report freedom of ATa, HRQoL, and AF related symptoms after 3.7 ±1.0 year follow-up in patients with persistent AF who were treated with the second generation CB ablation. Despite a considerable ATa recurrence rate (55%), AF related symptoms improved significantly upon CB ablation and HRQoL was high at follow-up. Only a small proportion (8%) of patients deteriorated into LS persistent AF and 75% of the patients did not report any AF at 5 year follow-up.

### Atrial tachyarrhythmia recurrences

A relevant decline in event-free survival has been reported 5–10 years after PVI [15, 16, 25–27]. Two previous studies, investigating outcomes after radiofrequency ablation, reported that only 29% and 32% of the patients where free of ATa after 5 and 10 years respectively [25, 26]. Neumann et al. described five years success after the first-generation CB in 53% of the patients with paroxysmal AF [27]. More recently, other authors report outcomes five years after ablation using the second-generation CB with event-free survival rates of 55% and 59%. These studies revealed that 60 and 61% of patients with paroxysmal AF were free of ATa compared to 32% and 52% of patients with persistent AF [15, 16]. Akkaya et al. reported ATa outcomes after a median follow-up of 33 [25–48] months in 281 patients with persistent AF who were treated with the second generation CB. These authors observed freedom of ATa in 63% of the patients [17]. Thus, the arrhythmia recurrence rate in our study is comparable with the above-mentioned previous findings, despite the somewhat variant duration of clinical follow-ups.

### Health-related quality-of-life

Besides the arrhythmia recurrence, our study emphasized on improvement of AF related symptoms and the HRQoL after PVI. Given the high AF recurrence rate consistently reported in previous studies as well as our data, PVI may is likely not a permanent cure for AF. Thus, it is reasonable to pay more attention to quality of life of patients treated with PVI [1, 3, 10, 12]. Previous studies suggested that PVI shows higher efficiency in improving HRQoL as compared to anti-arrhythmic drugs [1, 3]. To assess the HRQoL of patients, we adopted the AFEQT quality-of-life assessment tool that was developed to evaluate AF specific quality-of-life with a high sensitivity and specificity to detect AF related quality-of-life changes [23, 28]. At twelve months after PVI, the overall AFEQT score ranges from 82.9–89.5 points, consistently with previous findings from patients with paroxysmal and persistent AF [1, 28, 29]. The CABANA sub-study showed that the AFEQT score remained stable during a 60 month follow-up period in population consisted of patients both with paroxysmal and persistent AF without AF type-specific outcomes [1]. Thus, our study with 2–5 year follow-up specifically for persistent AF treated by the second generation CB may represent a more comprehensive data serving as an important addition in the field.

In addition, our study showed an notable improvement in AF related symptoms in 80% of the patients based on mEHRA scores that are significantly correlated with AFEQT score (Spearmans ρ -0.74; p < 0.001). Similarly, a previous study also have found a significant trend towards lower AFEQT scores in patients with a higher mEHRA score [24]. For example, patients with an mEHRA score of IIa had an AFEQT score of 70.9 ±19.8 compared to an AFEQT score of 58.3 ±17.3 in patients with mEHRA score of IIb [24]. These findings are in line with our data as we found that an AFEQT score > 75 represents mild to moderate symptoms (mEHRA I, IIa) and an AFEQT score ≤ 75 represents moderate to disabling symptoms (mEHRA IIb–IV).

### Arrhythmia burden

In this study, the primary outcome was met in 55% of the patients. However, a single recurrence, defined as an episode of ATa >30 seconds, might not fully represent procedural success. The CIRCA DOSE study randomized patients with paroxysmal AF to contact-force radiofrequency ablation, CB 2-minute or CB 4-minute ablation strategy, and all patients received an implantable loop recorder before the ablation. Overall, that study showed a > 99% reduction of atrial tachyarrhythmia burden but only 53% of the patients were free of any atrial tachyarrhythmia at 12 months [5]. In addition, the AFACT trial showed that patients with a single AF recurrence had a similar HRQoL compared to patients without a recurrence [30]. Others have reported an improvement of HRQoL in patients who had an ATa burden of less than 12 hours per month. Although our study does not present ATa burden, we do demonstrate that for each year of follow-up the large majority of patients were in sinus rhythm and that the average number of ECV in patients with an ATa recurrence significantly decreased after PVI. This suggests a reduction of burden and explains, despite the recurrence rate, the improvement of AF related symptoms and a high HRQoL.

### Study limitations

Despite the promising outcome showing a correlation between AF symptoms and quality-of-life, this study has several limitations. First, the design of this study was cross-sectional and non-randomized. Follow-up was performed in routine clinical cares and most of the data were collected retrospectively. To overcome this issue, we prospectively applied a seven-day Holter in all patients who did not have a CIED before the final follow-up visit. Secondly, AFEQT questionnaire were only filled-out at follow-up not before the PVI treatment. Therefore, we cannot report the change of HRQoL after CB ablation in these patients. We observed, however, improvement of AF related symptoms (decrease in mEHRA class) which strongly correlates to the AFEQT score. Lastly, all patients in this study were treated only with the second generation CB. The used ablation modality is based on operators’ discretion, and selection bias might be present.

## Conclusions

Intermediate-term follow-up after second generation CB PVI demonstrated that 45% of the patients with persistent AF were free of ATa recurrence by 2–5 years. At follow-up, however, HRQoL was high and AF related symptoms improved in 80% of the patients. More research is needed to understand the relation between recurrence frequency and duration, HRQoL and AF related symptoms, after PVI in patients with persistent AF.

## Supporting information

S1 Data CRYOpers data discription. (CSV)

S2 Data CRYOpers data. (CSV)
